# Diagnostic Accuracy of Shear Wave Elastography for the Assessment of Congestive Liver Disease in Patients With Structural Heart Disease

**DOI:** 10.1002/jcu.70283

**Published:** 2026-06-02

**Authors:** Edinéia Camargo Dziadzio, Maria Angelica Jungles Malikoski, Eduardo Henrique Bonotto, Sandra Monica Tochetto, Marco Stephan Lofrano‐Alves

**Affiliations:** ^1^ Post Graduate Program in Internal Medicine, Internal Medicine Department Federal University of Parana Curitiba Parana Brazil

**Keywords:** congestive hepatopathy, congestive liver disease, heart failure, liver stiffness, shear wave elastography

## Abstract

**Introduction:**

Shear wave elastography (SWE) is widely used to assess liver stiffness in chronic liver disease. Increased stiffness may also occur in congestive liver disease (CLD) due to hepatic venous congestion in patients with cardiovascular disease, but data on its diagnostic performance remain limited.

**Objectives:**

To evaluate the diagnostic performance of SWE for detecting CLD in outpatients with structural heart disease undergoing investigation for liver disease.

**Methods:**

Consecutive patients referred for SWE between 2018 and 2023 were retrospectively reviewed. CLD was defined by compatible liver imaging findings and structural heart disease on transthoracic echocardiography. Diagnostic performance was assessed using ROC analysis. Associations between liver stiffness and echocardiographic parameters were evaluated using correlation and partial correlation analyses, and determinants of liver stiffness were assessed using multiple linear regression.

**Results:**

About 88 patients were included (17 with CLD). Liver stiffness was higher in CLD compared with non‐CLD patients (8.4 [7.5–9.6] vs. 7.2 [6.5–8.2] kPa, *p* < 0.001). A cutoff > 7.54 kPa yielded an AUC of 0.757, with 81.2% sensitivity and 63.4% specificity. CLD was associated with right ventricular dilation and greater tricuspid regurgitation severity. Significant correlations were observed with right ventricular diameter, pulmonary artery systolic pressure, right atrial volume, and left atrial volume index. In multiple linear regression, CLD remained the only independent determinant of liver stiffness.

**Conclusions:**

SWE shows moderate diagnostic accuracy for detecting congestive hepatopathy in patients with structural heart disease. Liver stiffness appears to reflect systemic venous congestion rather than isolated hepatic structural disease.

## Graphical abstract

Shear wave elastography demonstrated moderate diagnostic accuracy for detecting congestive liver disease in patients with structural heart disease. Increased liver stiffness was associated with echocardiographic markers of venous congestion, supporting the concept that elastography may serve as a noninvasive marker of cardiohepatic interaction.

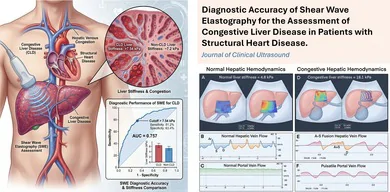

## 1 Introduction

Heart failure (HF) is a prevalent clinical syndrome characterized by structural or functional cardiac abnormalities leading to inadequate tissue perfusion and elevated intracardiac pressures. Beyond its cardiovascular impact, HF often induces multiorgan dysfunction, including hepatic impairment (McDonagh et al. 2022; Heidenreich et al. 2022). Congestive liver disease (CLD) arises from chronic elevation of central venous pressure, leading to sinusoidal dilation, centrilobular congestion, and ultimately fibrosis. The bidirectional relationship between cardiac and hepatic dysfunction—termed the cardiohepatic syndrome—carries important prognostic implications (Xanthopoulos et al. 2019).

Conventional diagnosis of CLD relies on clinical suspicion supported by imaging findings, laboratory markers, and invasive measurements, but these methods are either qualitative, operator‐dependent, or not feasible for serial monitoring. Shear wave elastography (SWE) is a noninvasive ultrasound‐based technique that quantifies liver stiffness, widely validated in chronic viral and metabolic liver diseases. While SWE correlates with portal hypertension and fibrosis severity in noncardiac hepatopathies, evidence on its diagnostic accuracy for CLD remains scarce (Fortea et al. 2020).

This study aimed to assess the diagnostic accuracy, sensitivity, and specificity of SWE for detecting CLD in patients with structural heart disease, to evaluate correlations with echocardiographic parameters, and to investigate its prognostic value.

## 2 Methods

### 2.1 Study Design and Population

This was a single‐center, observational, retrospective study conducted at the Hospital de Clínicas, Federal University of Paraná, Brazil. Consecutive outpatients who underwent their first liver SWE examination between 2018 and 2023 were identified through institutional databases.

Clinical and demographic data were obtained through review of electronic medical records. The study was approved by the local Research Ethics Committee (protocol no. 6.911.684), which waived the requirement for written informed consent due to the retrospective design and the use of previously recorded medical data. The study was registered in Plataforma Brasil (no. 75157223.5.0000.0096). All procedures were conducted in accordance with the principles of the Declaration of Helsinki, ensuring confidentiality and anonymity of patient information.

Patients were excluded if age was < 18 or > 80 years, if elastography images were not available, if acoustic windows were inadequate for elastography analysis, or if clinical data were incomplete in the medical record. Patients with Budd–Chiari syndrome or venous flow abnormalities related to thrombosis of major hepatic vessels were also excluded.

Patients were classified as having CLD when clinical suspicion was confirmed by Doppler ultrasound findings obtained concomitantly with SWE (Figure 1). CLD was supported by Doppler ultrasound findings consistent with hepatic venous congestion. These included marked pulsatility of the hepatic venous waveform with reduction or reversal of the systolic (S) wave, reflecting transmission of elevated right atrial pressure to the hepatic venous circulation. Additional supportive findings included dilation of the hepatic veins and inferior vena cava, and increased pulsatility of the portal vein waveform.

**FIGURE 1:**
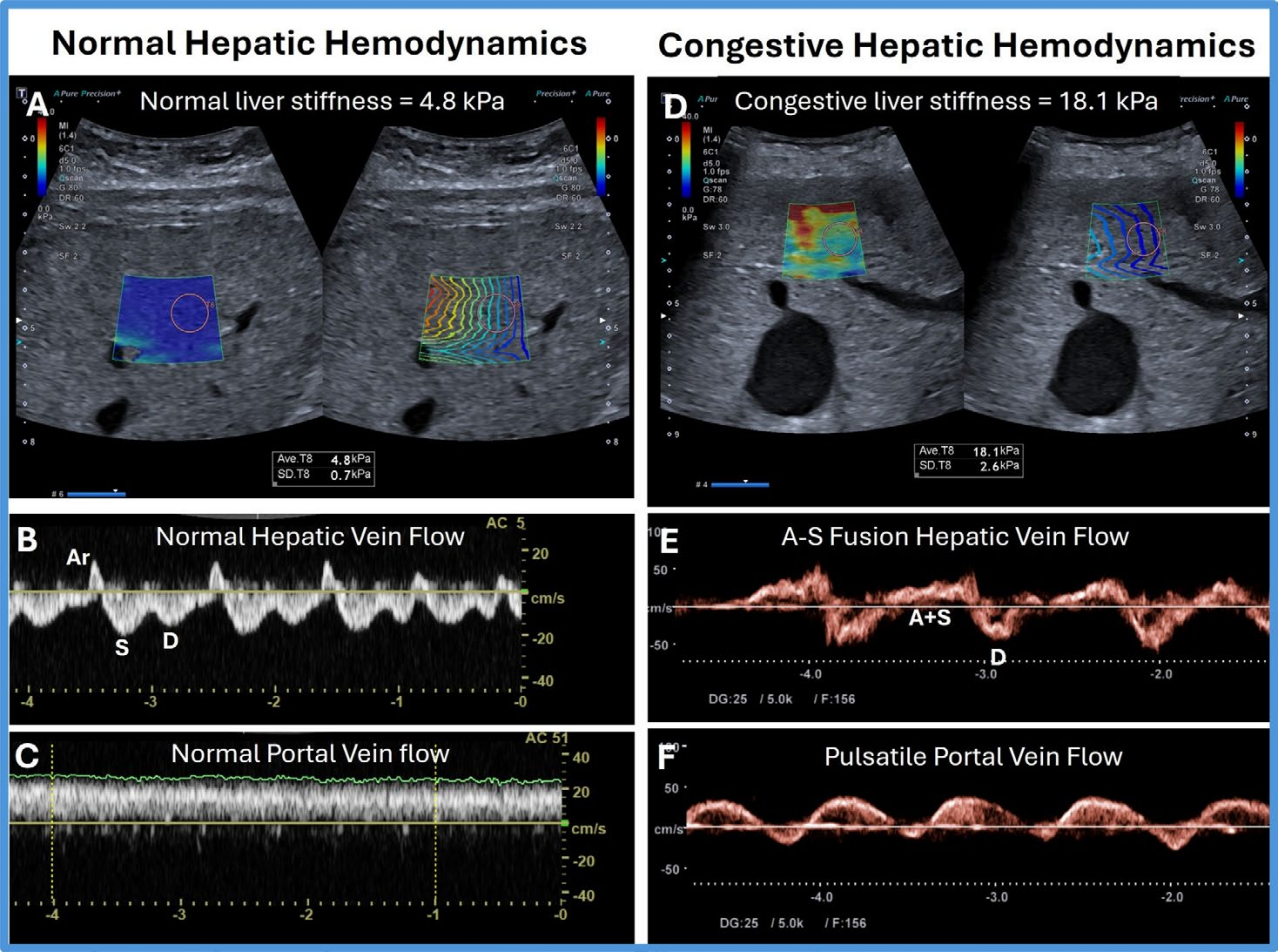
Shear wave elastography (SWE) and Doppler ultrasound findings in normal and congestive liver disease. (A–C) Representative findings in a normal subject. (A) Two‐dimensional SWE demonstrates normal liver stiffness (4.8 kPa). (B) Doppler waveform of the hepatic vein showing the normal triphasic pattern with systolic (S), diastolic (D), and atrial reversal (Ar) waves. (C) Portal vein Doppler waveform demonstrating continuous hepatopetal flow with minimal pulsatility. (D–F) Representative findings in a patient with congestive liver disease (CLD). (D) SWE color map and propagation map demonstrate markedly increased liver stiffness (18.1 kPa). (E) Hepatic vein Doppler waveform showing fusion of atrial and systolic components (A–S fusion), reflecting transmission of elevated right‐sided cardiac pressures. (F) Portal vein Doppler demonstrating increased pulsatility of portal flow, consistent with hepatic venous congestion.

These Doppler features were interpreted in conjunction with clinical data and the presence of structural heart disease on transthoracic echocardiography to establish the diagnosis of congestive hepatopathy. Additional imaging methods, including abdominal computed tomography or magnetic resonance imaging when available, and the presence of structural heart disease assessed by transthoracic echocardiography, were used to support the diagnosis. Final classification was based on an integrated assessment of imaging findings and clinical context, as recommended for the evaluation of congestive hepatopathy in patients with cardiovascular disease.

Structural heart disease was defined according to current echocardiographic guidelines as the presence of myocardial hypertrophy (left ventricular mass index > 115 g/m^2^ in men or > 95 g/m^2^ in women), reduced left ventricular ejection fraction (LVEF < 53%), regional wall motion abnormalities, right ventricular (RV) systolic dysfunction, or enlargement of cardiac chambers.

Patients with HF were classified according to New York Heart Association (NYHA) functional class (I–IV). Patients with chronic liver disease were classified using MELD and Child–Pugh scores and according to liver disease etiology (Peng et al. 2016). Clinical and laboratory data used for these classifications were obtained from medical consultations and laboratory tests performed within 1 month of the first SWE examination.

### 2.2 Shear Wave Elastography

Liver stiffness was measured in kilopascals (kPa) using a GE Logiq S8 ultrasound system equipped with two‐dimensional shear wave elastography (2D‐SWE). Measurements were obtained in the right hepatic lobe through an intercostal approach with patients in the supine or left lateral decubitus position and the right arm elevated (Figure 1). At least five valid measurements were acquired, and the median value was used for analysis. Measurements were performed during brief breath‐hold to minimize the influence of respiratory variations, as deep inspiration and the Valsalva maneuver can increase hepatic venous pressure and artificially alter liver stiffness measurements (Barr et al. 2020).

### 2.3 Echocardiographic Assessment

Transthoracic echocardiography was performed according to the recommendations of the American Society of Echocardiography and the European Association of Cardiovascular Imaging. Standard measurements included cardiac chamber dimensions, left ventricular ejection fraction (LVEF), RV basal diameter, RV systolic function, pulmonary artery systolic pressure (PASP), and left atrial volume index (LAVI). Right atrial volume, tricuspid regurgitation severity, and indices of diastolic function were also recorded when available (Lang et al. 2015; Nagueh et al. 2016).

### 2.4 Clinical Outcomes

The primary objective of the study was to evaluate the diagnostic performance of SWE for the identification of CLD. Secondary objectives included the investigation of associations between liver stiffness and echocardiographic markers of cardiovascular remodeling and systemic congestion, as well as exploratory evaluation of clinical outcomes. The composite outcome included all‐cause mortality, hospitalization for decompensated HF, hospitalization for acute coronary syndrome, hospitalization for decompensated liver disease, or major hepatic complications such as variceal bleeding. Clinical events were identified through retrospective review of electronic medical records.

### 2.5 Statistical Analysis

Continuous variables are presented as mean ± standard deviation or median with interquartile range (IQR), depending on distribution assessed by the Shapiro–Wilk test. Categorical variables are expressed as counts and percentages.

Comparisons between groups were performed using the Student's *t*‐test or Mann–Whitney U test for continuous variables and the chi‐square test or Fisher's exact test for categorical variables.

Diagnostic performance of SWE for identifying CLD was evaluated using receiver operating characteristic (ROC) analysis. The optimal cutoff value was determined based on the Youden index.

Associations between CLD and echocardiographic parameters were assessed using correlation analyses (Pearson or Spearman as appropriate). Partial correlation analyses were performed to evaluate the relationship between liver stiffness and CLD after adjustment for potential confounding variables.

To identify determinants of liver stiffness, multiple linear regression analysis was performed using SWE as the dependent variable. Independent variables included echocardiographic parameters reflecting cardiac remodeling and hemodynamic burden.

Event‐free survival was explored using Kaplan–Meier analysis with group comparisons performed using the log‐rank test; given the limited number of events, these analyses were considered exploratory. All statistical analyses were performed using MedCalc software (version 23.1). A two‐tailed *p* value < 0.05 was considered statistically significant.

## 3 Results

### 3.1 Study Population

Initially, 2553 SWE examinations were identified. After excluding duplicate exams and cross‐matching patients who underwent cardiologic evaluation with echocardiography within 6 months of the first SWE, 153 patients were selected. About 65 were excluded due to insufficient medical record data, leaving 88 patients for analysis: 71 without CLD and 17 with CLD (Figure 2).

**FIGURE 2:**
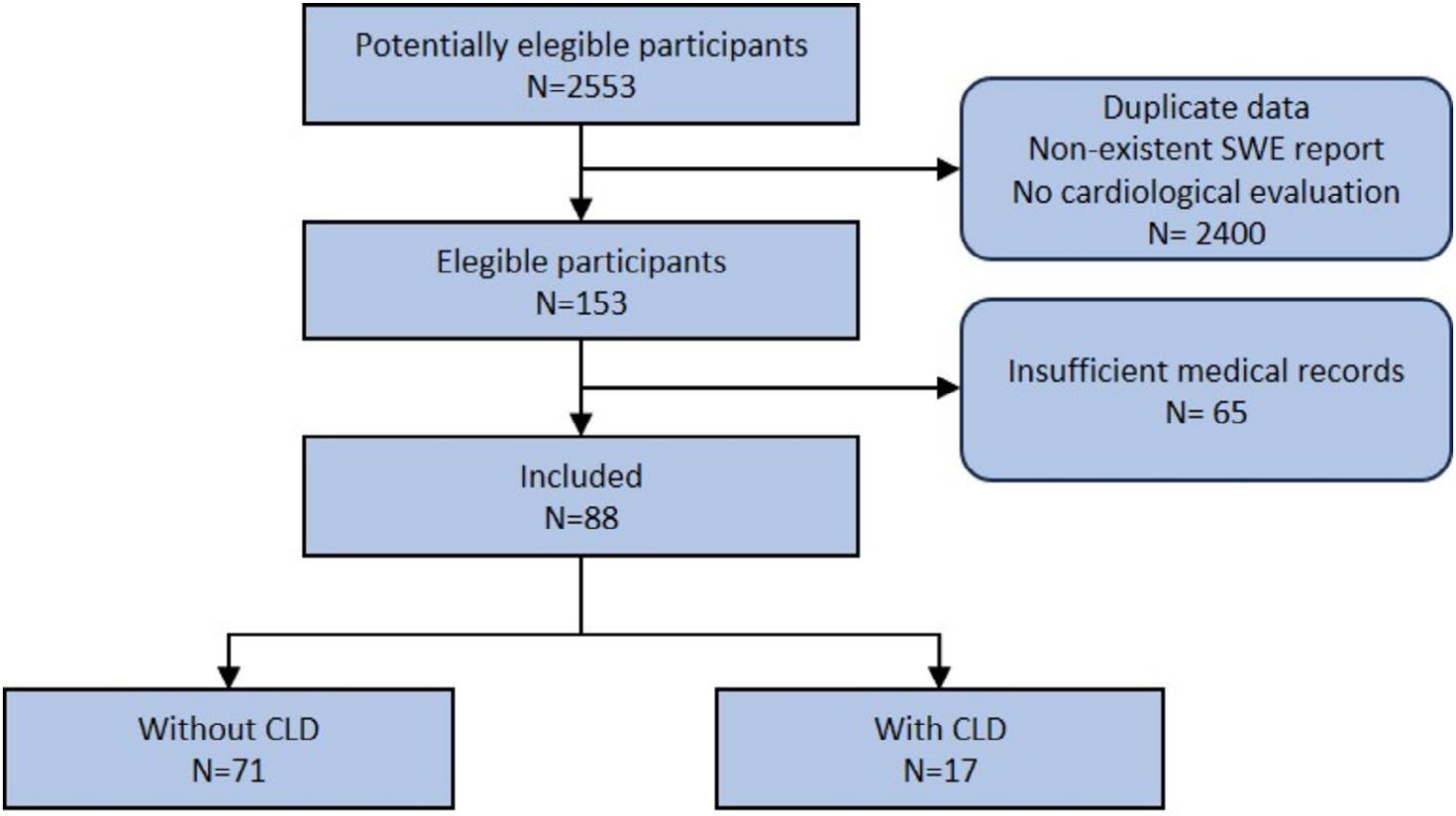
Patient selection flowchart. Flow diagram depicting patient selection. Among 2553 potentially eligible shear wave elastography (SWE) examinations, duplicate studies, missing SWE reports, and absence of cardiological evaluation were excluded. After exclusion due to insufficient medical records, 88 patients were included in the final analysis, comprising 71 patients without congestive liver disease (CLD) and 17 patients with CLD.

Baseline characteristics are summarized in Table 1. The median age was 64 years, mean BMI 29.0 kg/m^2^, 64% were female, 29.5% had cirrhosis, and 88.6% hepatic steatosis. Cardiovascular risk factors included hypertension (82.9%), dyslipidemia (73.9%), diabetes (46.5%), smoking (36.0%), and obesity (79.8%). Coronary artery disease was present in 26.1%.

**TABLE 1:** Comparison of clinical and demographic variables in patients with and without congestive liver disease (CLD).

Variable	All (*n* = 88)	No CLD (*n* = 71)	CLD (*n* = 17)	*p*
Age (years)	64.0 (57.0–70.0)	64.0 (57.0–70.0)	59 (50.2–71.0)	NS
BMI (kg/m^2^)	29.0 (25.7–31.6)	29.4 (26.5–32.0)	25.3 (21.8–31.1)	NS
Female sex	69 (64%)	47 (66.2%)	13 (76.5%)	NS
Cirrhosis	26 (29.5%)	17 (27.4%)	9 (64.3%)	0.009
CHILD classification				0.006
A	68 (77.3%)	56 (78.9%)	12 (70.6%)	
B	3 (3.4%)	1 (1.4%)	2 (11.7%)	
C	0 (0%)	0 (0%)	0 (0%)	
Etiology				< 0.0001
Virus	15 (17%)	14 (19.7%)	1 (5.9%)	
Alcohol	9 (10.2%)	9 (12.7%)	0 (0%)	
MASLD	40 (45.4%)	35 (49.3%)	5 (29.4%)	
Autoimmune	3 (3.4%)	3 (4.2%)	0 (0%)	
Primary biliary	2 (2.2%)	2 (2.8%)	0 (0%)	
Right‐sided HF	6 (6.8%)	0 (0%)	6 (35.3%)	
Other	2 (2.3%)	5 (7.0%)	3 (17.7%)	
Steatosis	78 (88.6%)	64 (90.1%)	14 (82.3%)	NS
CV risk factors				
Hypertension	73 (82.9%)	59 (83.1%)	14 (82.3%)	NS
DM	41 (46.5%)	33 (46.4%)	8 (47.0%)	NS
Dyslipidemia	65 (73.9%)	53 (74.6%)	12 (70.5%)	NS
Smoking	32 (36.0%)	25 (35.2%)	7 (41.1%)	NS
Obesity	71 (79.8%)	60 (84.5%)	11 (64.7%)	NS
CAD	23 (26.1%)	13 (18.3%)	9 (52.9%)	0.005
Heart failure				0.059
NYHA I	63 (71.6%)	53 (74.6%)	10 (58.8%)	
NYHA II	18 (20.5%)	13 (18.3%)	5 (29.4%)	
NYHA III–IV	7 (7.9%)	4 (5.6%)	3 (17.6%)	

Abbreviations: BMI, body mass index; CAD, coronary artery disease; CLD, congestive liver disease; CV, cardiovascular; DM, diabetes mellitus; MASLD, metabolic dysfunction‐associated steatotic liver disease; MELD, model for end‐stage liver disease; NS, not significant; NYHA, New York Heart Association functional class.

Cirrhosis was significantly more frequent in the CLD group. Liver disease etiology also differed, with MASLD and viral hepatitis predominating in non‐CLD patients, while right HF and MASLD were more common in CLD. Patients with CLD more frequently presented Child–Pugh class B, coronary artery disease, and NYHA III–IV functional class, although most patients overall were NYHA I (71.6%), followed by NYHA II (20.5%) and NYHA III/IV (7.9%).

### 3.2 Liver Stiffness and Diagnostic Performance

Liver stiffness measured by SWE‐PV was significantly higher in the CLD group compared with the non‐CLD group (8.4 kPa [IQR 7.5–9.6] vs. 7.2 kPa [IQR 6.5–8.2], *p* < 0.001) (Table 2). ROC analysis demonstrated moderate diagnostic performance for detecting CLD (Figure 3). Using a cutoff > 7.54 kPa, the AUC was 0.757, with 81.2% sensitivity and 63.4% specificity.

**TABLE 2:** Comparison of elastography and echocardiography variables in patients with and without congestive liver disease.

Variable	All (*n* = 88)	No CLD (*n* = 71)	CLD (*n* = 17)	*p*
Elastography				
Stiffness^a^ (kPa)	7.4 (6.5–8.3)	7.2 (6.5–8.2)	8.4 (7.5–9.6)	< 0.001
METAVIR^b^				< 0.001
F0/F1	21 (23.8%)	20 (28.1%)	1 (5.8%)	
F2/F3	51 (58.0%)	43 (60.5%)	8 (47%)	
F4	16 (18.2%)	8 (11.2%)	8 (47%)	
Echocardiography				
SPT (mm)	9.0 (9.0–11.0)	9.0 (9.0–10.0)	9.0 (9.0–11.0)	NS
PP (mm)	9.0 (8.0–10.0)	9.0 (8.0–10.0)	9.0 (8.7–9.2)	NS
LVDD (mm)	46.0 (42.0–50.0)	46.0 (42.0–49.7)	47.0 (44.0–51.0)	NS
LVMI (mg/m^2^)	77.5 (66.1–99.7)	76.1 (64.8–93.7)	91.1 (73.0–104.5)	NS
LVEF (%)	66.0 (60.7–69.2)	66.0 (61.2–69.0)	64.0 (61.2–70.5)	NS
LAVI (mL/m^2^)	30.4 (26.0–36.0)	29.0 (25.2–35.0)	37.0 (31.5–52.5)	0.002
Septal e' (cm/s)	6.2 (4.7–7.6)	6.2 (4.7–7.4)	4.8 (4.8–10.6)	NS
Lateral e' (cm/s)	8.7 (6.7–9.8)	8.6 (6.7–9.7)	6.1 (6.5–17.0)	NS
E/A ratio	0.8 (0.6–1.0)	0.8 (0.6–1.0)	0.7 (0.5–1.5)	NS
E/e' mean ratio	9.2 (7.3–11.1)	9.6 (7.5–12.0)	8.4 (5.8–10.1)	NS
Diastolic function				NS
Normal	26 (29.5%)	23 (32.3%)	3 (17.6%)	
Grade I	30 (34.0%)	21 (29.5%)	9 (52.9%)	
Grade II	10 (11.3%)	9 (12.6%)	0 (0%)	
Grade III	0 (0%)	0 (0%)	0 (0%)	
Indeterminate	22 (31%)	18 (25.3%)	5 (29.4%)	
PASP (mmHg)	31.5 (27.0–41.5)	31.0 (26.7–41.0)	35.0 (39.5–51.2)	NS
Basal RV (A4C) diameter (mm)	35.0 (35.0–37.0)	35.0 (35.0–36.5)	35.0 (35.0–44.0)	NS
FAC (%)	30.0 (22.0–44.5)	36.5 (22.0–51.0)	30.0 (22.0–38.0)	NS
TAPSE (mm)	21.0 (19.0–24.0)	21.0 (19.0–24.2)	20.0 (17.5–22.0)	NS

Abbreviations: A, peak late diastolic transmitral flow velocity after atrial contraction by pulsed Doppler; CLD, congestive liver disease; E, peak early diastolic transmitral flow velocity by pulsed Doppler; E/e' mean ratio, ratio of E velocity to the mean of septal and lateral e' velocities; FAC, fractional area change; Lateral e', peak early diastolic mitral annular velocity at the lateral side by tissue Doppler; LAVI, left atrial volume index; LV, left ventricle; LVDD, left ventricle diastolic diameter; LVEF, left ventricular ejection fraction; LVMI, left ventricle mass index; PASP, pulmonary artery systolic pressure; PP, posterior wall thickness; RV basal A4C, basal right ventricular diameter at apical four‐chamber view; Septal e', peak early diastolic mitral annular velocity at the septal side by tissue Doppler; SPT, septal thickness; TAPSE, tricuspid annular plane systolic excursion.

^a^ Assessed by shear wave elastography.

^b^ METAVIR classification (“meta‐analysis of histological data in viral hepatitis”) based on shear wave velocity cut‐off values.

**FIGURE 3:**
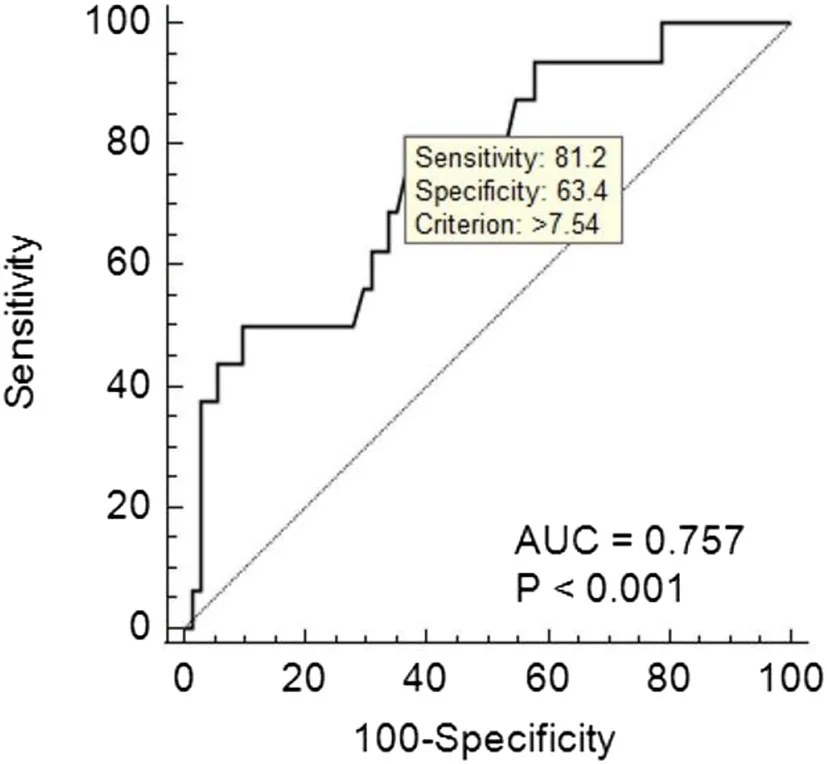
Diagnostic accuracy of shear wave elastography for congestive liver disease. Receiver operating characteristic (ROC) curve illustrating the diagnostic performance of SWE‐derived liver stiffness for the identification of congestive liver disease. A cutoff value > 7.54 kPa yielded a sensitivity of 81.2% and specificity of 63.4%, with an area under the curve (AUC) of 0.757 (*p* < 0.001).

### 3.3 Echocardiographic Findings

LAVI was significantly higher in patients with CLD. Although median PASP and RV dimensions did not differ significantly between groups, interquartile analysis showed relevant differences. In the CLD group, the 75th percentile of PASP exceeded 51 mmHg, and the 75th percentile of basal RV diameter exceeded 44 mm, above the reference limit of 42 mm.

### 3.4 Frequency of Abnormal Right Ventricular Parameters

RV dilation was significantly more frequent in patients with CLD (30.8% vs. 6.7%, *p* = 0.012). In contrast, abnormal PASP and reduced TAPSE frequencies did not differ significantly between groups. Tricuspid regurgitation severity differed significantly between groups (*χ* ^2^ = 10.2, *p* = 0.036), with moderate‐to‐severe regurgitation (≥ grade 2) more common in the CLD group.

### 3.5 Correlation Analyses

Congestive hepatopathy showed significant correlations with markers of cardiovascular remodeling and congestion, including basal RV diameter, PASP, right atrial volume, LAVI, and presence of cirrhosis (Table 3).

**TABLE 3:** Hemodynamic and structural correlates of congestive liver disease.

Variable	Correlation coefficient (*r*)	*p*
Basal RV diameter	0.323	0.005
Pulmonary artery systolic pressure	0.282	0.042
Right atrial volume	0.268	0.018
Left atrial volume index	0.387	< 0.001
Presence of cirrhosis	0.301	0.008

*Note:* Correlation coefficients represent the association between each variable and the presence of congestive liver disease.

Partial correlation analyses confirmed that the association between SWE‐derived liver stiffness and CLD remained significant after adjustment for several variables, including basal RV diameter (*r* = 0.459, *p* < 0.001), right atrial volume (*r* = 0.433, *p* < 0.001), PASP (*r* = 0.413, *p* = 0.003), LAVI (*r* = 0.412, *p* < 0.001), NYHA class (*r* = 0.434, *p* = 0.004), and age (*r* = 0.426, *p* < 0.001). The association remained significant after adjustment for cirrhosis (*r* = 0.258, *p* = 0.026), suggesting that liver stiffness reflects systemic venous congestion beyond isolated cardiac or hepatic structural changes.

### 3.6 Determinants of Liver Stiffness

Multiple linear regression analysis was performed using SWE as the dependent variable. Independent variables included basal RV diameter, LAVI, LVEF, diastolic function grade, tricuspid regurgitation severity, and CLD. CLD remained the only independent predictor of liver stiffness (*β* = 2.20 kPa, 95% CI 0.56–3.84, *p* = 0.009). Echocardiographic variables were not independently associated with SWE values (Table S1).

### 3.7 Exploratory Outcome Analysis

During follow‐up, 11 adverse clinical events were identified through retrospective medical record review, including hospitalizations for cardiovascular or hepatic causes and all‐cause mortality. In exploratory analyses, patients with CLD showed lower event‐free survival compared with those without CLD (log‐rank *p* = 0.003) (Figure S1).

Events included hospitalization for chest pain of unclear etiology (*n* = 1), decompensated HF (*n* = 3), acute coronary syndrome including myocardial infarction (*n* = 2), HF due to aortic regurgitation requiring valve replacement (*n* = 1), and hepatic decompensation with upper gastrointestinal bleeding from esophageal varices (*n* = 1). Three deaths occurred: one probable progression of cirrhosis without documented cause, one due to variceal bleeding, and one due to cardioembolic stroke complicated by hemorrhagic transformation and sepsis.

These events reflect both cardiovascular and hepatic complications. Given the retrospective design, events were identified through chart review rather than formal adjudication, and outcome analyses were considered exploratory (Table S2).

## 4 Discussion

### 4.1 Principal Findings

In this study, we demonstrated that SWE provides moderate diagnostic accuracy for identifying CLD in patients with structural heart disease, with an area under the ROC curve (AUC) of 0.757. The optimal SWE cutoff of > 7.54 kPa yielded a sensitivity of 81.2% and specificity of 63.4%. In addition, patients with CLD showed a higher prevalence of RV remodeling and more advanced tricuspid regurgitation.

Correlation analyses further demonstrated significant associations between congestive hepatopathy and markers of cardiovascular remodeling and filling pressures, including RV diameter, PASP, right atrial volume, and LAVI. Importantly, the association between SWE‐derived liver stiffness and CLD remained significant after adjustment for several cardiovascular variables and after controlling for the presence of cirrhosis. Together, these findings suggest that hepatic stiffness reflects systemic venous congestion rather than simply mirroring isolated hepatic structural abnormalities.

### 4.2 Pathophysiological Mechanisms

The relationship between HF and liver disease has been increasingly recognized as part of the broader concept of cardiohepatic interaction. Chronic elevation of right‐sided filling pressures leads to passive hepatic congestion, sinusoidal dilatation, and progressive hepatic dysfunction. In this setting, increased hepatic stiffness may arise not only from fibrosis but also from venous congestion and elevated hepatic venous pressure (Xanthopoulos et al. 2019; Fortea et al. 2020).

Our findings support this mechanism. Patients with CLD demonstrated a higher prevalence of RV dilation and greater severity of tricuspid regurgitation, both of which contribute to elevated right atrial pressure and systemic venous congestion. Furthermore, the observed correlations between hepatic stiffness and right‐sided hemodynamic markers reinforce the link between cardiac remodeling and hepatic involvement.

Previous studies have reported similar findings. Ohara et al. demonstrated that liver stiffness measured by elastography correlates with right atrial pressure and predicts adverse outcomes in patients with HF (Ohara et al. 2020). Likewise, other studies have shown that elastography parameters of the liver or spleen are associated with congestion severity and clinical outcomes in HF populations (Nakayama et al. 2022; Misaka et al. 2023). These observations support the hypothesis that hepatic stiffness can serve as a non‐invasive surrogate marker of systemic venous congestion.

Importantly, the persistence of the association between SWE and CLD even after adjustment for cirrhosis suggests that elastography captures hemodynamic congestion beyond intrinsic hepatic structural disease. This finding is consistent with the understanding that elastography measurements may reflect both fibrosis and transient changes in hepatic venous pressure.

Also, we must acknowledge that hepatic manifestations of CLD may also reflect heterogeneous perfusion disturbances in addition to passive venous congestion. In some cases, focal hepatic abnormalities related to altered perfusion may mimic true focal liver lesions on imaging. Such pseudolesions have been described in the literature and may require further characterization using contrast‐enhanced ultrasound or cross‐sectional imaging (CT or MRI). These findings highlight that imaging manifestations of congestive hepatopathy may be nonspecific and should be interpreted within the broader clinical and hemodynamic context (Guarino et al. 2015). In contrast to focal perfusion abnormalities, the elastographic findings observed in our study reflect diffuse hepatic stiffness, which is more directly linked to systemic venous congestion.

An important consideration when interpreting elastography findings in this context is that liver stiffness is influenced not only by structural fibrosis but also by hemodynamic factors. Experimental and clinical studies have demonstrated that hepatic stiffness may increase in response to elevated central venous and hepatic venous pressures, reflecting passive congestion rather than irreversible fibrotic remodeling. Consequently, elastography measurements in patients with cardiovascular disease should be interpreted within the clinical and hemodynamic context. In this scenario, increased stiffness may represent a dynamic marker of systemic venous congestion rather than a specific indicator of intrinsic liver disease.

### 4.3 Clinical Implications

Our findings align with the growing literature affirming SWE as a valuable, non‐invasive method for evaluating chronic liver diseases (Barr et al. 2020; Varbobitis et al. 2016; Targher et al. 2025). A WFUMB guideline underlines SWE's role in diagnosing and managing chronic liver disease, citing its robust reproducibility and diagnostic performance across fibrosis stages (Ferraioli et al. 2024a, 2024b). Moreover, a 2025 meta‐analysis on 2D‐SWE in metabolic‐associated steatotic liver disease (MASLD)—formerly NAFLD—reported summary AUCs ranging from 0.82 to 0.89 across fibrosis stages F1–F4, with optimized kPa cutoffs proposed (Xu et al. 2025). These data suggest that SWE's diagnostic potential may generalize well across chronic liver conditions, including our context of congestive hepatopathy in HF.

Our results extend these observations to the context of congestive hepatopathy. The SWE cutoff identified in this study (7.54 kPa) was lower than thresholds typically reported for advanced fibrosis, likely reflecting the predominant contribution of venous congestion rather than fibrosis in this population.

From a clinical perspective, non‐invasive identification of congestive hepatopathy may help improve the integrated assessment of patients with structural heart disease or HF. Detection of hepatic congestion may reflect advanced hemodynamic compromise and could contribute to risk stratification or monitoring of disease progression.

In exploratory analyses, patients with CLD also showed a higher incidence of adverse clinical events, including cardiovascular and hepatic complications. Although the number of events was limited, these findings are consistent with prior studies showing that increased liver stiffness is associated with worse outcomes in HF populations (Nakayama et al. 2022; Misaka et al. 2023).

### 4.4 Study Limitations

Some limitations should be acknowledged. First, this was a retrospective single‐center study with a relatively small sample size, which may limit statistical power and generalizability. Second, liver biopsy was not performed; however, histologic confirmation is rarely feasible in patients with HF and suspected congestive hepatopathy. Third, SWE measurements may be influenced by operator technique and hemodynamic conditions at the time of acquisition.

In addition, outcome analyses were exploratory because of the limited number of events and the retrospective identification of outcomes from medical records without formal adjudication. Finally, we did not evaluate shear wave dispersion, which may provide additional information for distinguishing congestion from hepatic fibrosis.

## 5 Conclusions

SWE provides a practical and non‐invasive tool with moderate diagnostic accuracy for detecting congestive hepatopathy in patients with structural heart disease. Liver stiffness appears to reflect systemic venous congestion and cardiovascular remodeling rather than isolated hepatic structural disease. These findings support the potential role of elastography as part of an integrated cardiohepatic assessment, although larger prospective studies are needed to further define its clinical applications.

## Author Contributions

**M.S.L**‐.**A.:** conception and design of the study, analysis and interpretation of data, revising it critically for important intellectual content, final approval of the version to be submitted. **E.C.D**. and **M.A.J.M.:** data acquisition, analysis and interpretation of data, drafting the article, final approval of the version to be submitted. **E.H.B.:** revising it critically for important intellectual content, final approval of the version to be submitted. **S.M.T.:** data acquisition, revising it critically for important intellectual content, final approval of the version to be submitted.

## Funding

The authors have nothing to report.

## Disclosure

The manuscript has not been published previously and is not under consideration for publication elsewhere. All authors have approved the final version of the manuscript.

## Ethics Statement

The study was approved by the local Research Ethics Committee, with a waiver of informed consent due to its retrospective design and was conducted in accordance with the Declaration of Helsinki.

## Conflicts of Interest

The authors declare no conflicts of interest.

## Supporting information


**Figure S1:** Exploratory Kaplan–Meier analysis of event‐free survival according to the presence of congestive liver disease. Because of the limited number of events, results should be interpreted as hypothesis‐generating.

**Table S1:** Determinants of liver stiffness (SWE) by multiple linear regression.

**Table S2:** Description and adjudication of clinical events.

## Data Availability

The data that support the findings of this study are available from the corresponding author upon reasonable request.
