# Supplementary material for: Diagnostic Accuracy of Shear Wave Elastography for the Assessment of Congestive Liver Disease in Patients With Structural Heart Disease
Source: J Clin Ultrasound. 2026 Jun 2;54(8):1867–75. doi: 10.1002/jcu.70283 (PMC13641328; doi:10.1002/jcu.70283)
Supplement: Supplementary file 1 [file JCU-54-1867-s001.docx]

**Supplementary Figure S1.** Exploratory Kaplan–Meier analysis of event-free survival according to the presence of congestive liver disease. Because of the limited number of events, results should be interpreted as hypothesis-generating.
