# Supplementary material for: Diagnostic Accuracy of Shear Wave Elastography for the Assessment of Congestive Liver Disease in Patients With Structural Heart Disease
Source: J Clin Ultrasound. 2026 Jun 2;54(8):1867–75. doi: 10.1002/jcu.70283 (PMC13641328; doi:10.1002/jcu.70283)
Supplement: Supplementary file 2 [file JCU-54-1867-s002.docx]

Supplementary table S1. Determinants of liver stiffness (SWE) by multiple linear regression.

| **Variable** | **β coefficient (kPa)** | **95% CI** | **p value** |
| --- | --- | --- | --- |
| Congestive liver disease | 2.20 | 0.56 to 3.84 | 0.009 |
| Basal RV diameter | -0.079 | -0.232 to 0.073 | 0.300 |
| LAVI | -0.021 | -0.081 to 0.039 | 0.475 |
| LVEF | 0.022 | -0.038 to 0.082 | 0.470 |
| Diastolic dysfunction grade | 0.378 | -0.479 to 1.234 | 0.379 |
| Tricuspid regurgitation grade | 0.705 | -1.337 to 2.748 | 0.490 |

Dependent variable: liver stiffness measured by shear wave elastography (kPa).
