# Supplementary material for: Diagnostic Accuracy of Shear Wave Elastography for the Assessment of Congestive Liver Disease in Patients With Structural Heart Disease
Source: J Clin Ultrasound. 2026 Jun 2;54(8):1867–75. doi: 10.1002/jcu.70283 (PMC13641328; doi:10.1002/jcu.70283)
Supplement: Supplementary file 3 [file JCU-54-1867-s003.docx]

Supplementary Table S2 – Description and adjudication of clinical events

| **Patient** | | **Event Type** | **Clinical Description** | **Cardiovascular or Hepatic** |
| --- | --- | --- | --- | --- |
| 1 | Hospitalization | | Chest pain of unclear etiology | Cardiovascular |
| 2 | Death | | Undetermined cause, probable progression of cirrhosis | Hepatic |
| 3 | Hospitalization | | Hepatic decompensation with upper gastrointestinal bleeding due to esophageal varices | Hepatic |
| 4 | Death | | Upper gastrointestinal bleeding due to esophageal varices | Hepatic |
| 5 | Hospitalization | | Decompensated heart failure (profile B) | Cardiovascular |
| 6 | Hospitalization | | Decompensated heart failure (NYHA III) with atypical chest pain | Cardiovascular |
| 7 | Hospitalization | | Decompensated heart failure (profile B) | Cardiovascular |
| 8 | Hospitalization | | Myocardial infarction treated with coronary stenting | Cardiovascular |
| 9 | Death | | Cardioembolic stroke complicated by hemorrhagic transformation and sepsis | Cardiovascular |
| 10 | Hospitalization | | Non-ST elevation myocardial infarction | Cardiovascular |
| 11 | Hospitalization | | Heart failure due to severe aortic regurgitation requiring valve replacement | Cardiovascular |
